# Type 2 Neural Progenitor Cell Activation Drives Reactive Neurogenesis after Binge-Like Alcohol Exposure in Adolescent Male Rats

**DOI:** 10.3389/fpsyt.2017.00283

**Published:** 2017-12-15

**Authors:** Chelsea R. Geil Nickell, Hui Peng, Dayna M. Hayes, Kevin Y. Chen, Justin A. McClain, Kimberly Nixon

**Affiliations:** ^1^Department of Pharmaceutical Sciences, University of Kentucky, Lexington, KY, United States

**Keywords:** neurogenesis, neural stem cell, ethanol, adolescence, alcohol use disorders

## Abstract

Excessive alcohol consumption during adolescence remains a significant health concern as alcohol drinking during adolescence increases the likelihood of an alcohol use disorder in adulthood by fourfold. Binge drinking in adolescence is a particular problem as binge-pattern consumption is the biggest predictor of neurodegeneration from alcohol and adolescents are particularly susceptible to the damaging effects of alcohol. The adolescent hippocampus, in particular, is highly susceptible to alcohol-induced structural and functional effects, including volume and neuron loss. However, hippocampal structure and function may recover with abstinence and, like in adults, a reactive burst in hippocampal neurogenesis in abstinence may contribute to that recovery. As the mechanism of this reactive neurogenesis is not known, the current study investigated potential mechanisms of reactive neurogenesis in binge alcohol exposure in adolescent, male rats. In a screen for cell cycle perturbation, a dramatic increase in the number of cells in all phases of the cycle was observed at 7 days following binge ethanol exposure as compared to controls. However, the proportion of cells in each phase was not different between ethanol-exposed rats and controls, indicating that cell cycle dynamics are not responsible for the reactive burst in neurogenesis. Instead, the marked increase in hippocampal proliferation was shown to be due to a twofold increase in proliferating progenitor cells, specifically an increase in cells colabeled with the progenitor cell marker Sox2 and S-phase (proliferation) marker, BrdU, in ethanol-exposed rats. To further characterize the individual subtypes of neural progenitor cells (NPCs) affected by adolescent binge ethanol exposure, a fluorescent quadruple labeling technique was utilized to differentiate type 1, 2a, 2b, and 3 progenitor cells simultaneously. At one week into abstinence, animals in the ethanol exposure groups had an increase in proliferating type 2 (intermediate progenitors) and type 3 (neuroblast) progenitors but not type 1 neural stem cells. These results together suggest that activation of type 2 NPCs out of quiescence is likely the primary mechanism for reactive hippocampal neurogenesis following adolescent alcohol exposure.

## Introduction

Alcohol use disorders (AUDs) remain a significant public health problem. Nearly 14% of the USA population meet the DSM-V diagnostic criteria for an AUD in any given year which translates into a life-time prevalence of 29% (1). AUDs often originate with experimentation with alcohol in adolescence, defined as ages 10–19 (2, 3). Indeed, DSM-IV based rates of AUDs in adolescence (~6%) were remarkably similar to that in adults [8.5% (4–7)]. Although rates of adolescent drinking have steadily declined over the last two decades (8), they are still high. For example, over 60% of adolescents report having consumed alcohol by 12th grade and more critically 5.7% (8th graders) to 37.3% (12th graders) have been drunk in the last year (8). Of those adolescents who drink alcohol, over half of them drink in a binge pattern, defined as greater than four (females) or five (males) drinks in a 2 h period (9, 10). Unfortunately, binge pattern drinking is associated with damage to the CNS (11) and adolescents show more degenerating neurons in corticolimbic regions than adults following binge/bender-like alcohol exposure in animal models (12). The adolescent’s greater susceptibility to alcohol-induced neurodegeneration may explain why hippocampal pathology has been observed in human adolescents with AUDs despite only a few years of drinking (13–16).

Drinking in young adolescence increases the risk of developing an AUD fourfold versus drinking onset at age 18 and older (17), which suggests that there are significant developmental differences in the effects of alcohol on the brain (16, 18–21). This heightened risk is due to a combination of several factors. Adolescence is a dynamic time for brain development, especially in frontal, cortical, and limbic behavioral control centers (22–24). Neurological immaturity coincides with increased risk taking, novelty seeking, and a reduced responsiveness to the sedative and motor impairing effects of alcohol intoxication [e.g., (25, 26)] that essentially create the “perfect storm” to drive excessive alcohol intake during adolescence (19, 21). The adolescent hippocampus, in particular, shows greater susceptibility to a host of negative effects resulting from excessive alcohol consumption including those from the intoxicating effects of alcohol as well as from the consequences of prior alcohol exposure (27–31). Human adolescents who meet criteria for an AUD demonstrate impairments on hippocampal-dependent tasks (32–34), which is in agreement with observations of reduced hippocampal volumes [(13–15); see also (35) for review]. Animal models of the consequences of adolescent alcohol consumption also demonstrate behavioral impairments on hippocampal-dependent tasks (36, 37), and have helped elucidate the underlying neurobiology, likely impairments in hippocampal structure and function (12, 27, 31, 38–40). However, others have seen only transient [e.g., (41)] or no effect (42) of prior alcohol exposure on hippocampal-dependent learning and memory behavior in adolescents.

The hippocampus is one of the few regions of the brain that contains a pool of neural stem cells (NSCs) that produce new neurons throughout the life of the organism (43–45). NSCs, located along the subgranular zone (SGZ), are now well accepted to produce granule cell neurons that contribute to hippocampal structure and function (45–50). The birth of new neurons is comprised of four main processes: cell proliferation, differentiation, migration, and survival/integration. Newly born neurons originate from a population of radial glia-like NSCs [type 1; (44)]. Type 1 NSCs self-renew by dividing asymmetrically to give rise to a daughter NSC and a daughter intermediate progenitor cell with glial (type 2a) or neuronal (type 2b) phenotypes, that then become a more lineage-committed neuroblast [type 3; reviewed in Ref. (45)]. Neuroblasts then migrate into the granule cell layer, extend axons and dendrites and become integrated as part of the hippocampal circuitry as they mature (45). Alcohol affects each of these processes depending on the timing (age), dose, duration, and pattern of exposure (51–53).

In animal models of AUDs, alcohol-induced neurodegeneration and recovery of hippocampal structure and function corresponds to a similar pattern in alcohol-induced effects on NSCs and adult neurogenesis [reviewed in Refs. (52–54)]. Specifically, alcohol intoxication inhibits NSC proliferation and adult neurogenesis in a duration-dependent and blood ethanol concentration (BEC)-dependent manner (55–63) while a rebound or compensatory effect on adult neurogenesis is observed during withdrawal and abstinence (64–68). Indeed, within the first several days of abstinence there is a striking burst in cell proliferation along the SGZ that results in a significant increase in newborn neurons in both adult and adolescent models of AUDs (64, 66–69). This reactive neurogenesis has been observed in other acutely damaging events such as traumatic brain injury (70), ischemia (71–73), and seizure (74, 75). Recent work describes that reactive NSC proliferation is due to stem cell activation in rodent models of traumatic brain injury (76) and alcohol dependence in adults.^1^ Specifically, an increase in the number of neural progenitor cells (NPCs) and proliferating NPCs was observed, suggesting an expansion of the stem cell pool (see text footnote 1). This expansion appears to be due, in part, to more type 1 NSCs recruited out of quiescence at 7 days of abstinence to help drive this reactive neurogenesis effect in adult rats (see text footnote 1). However, findings in adults or adult models do not necessarily generalize to adolescents. For example, the adolescent brain shows more profound and aberrant effects of alcohol on this reactive, adult neurogenesis phenomenon (67). In adolescent rats after alcohol dependence (the 4-day binge model), newborn neurons are observed in ectopic locations (67) and increases in the NSC pool have been observed immediately following the last dose of alcohol in adolescent rats but not adults (77). Therefore, due to these significant age differences in alcohol-induced reactive neurogenesis, we investigated the mechanism of reactive hippocampal neurogenesis in adolescent male rats after the 4-day binge model of alcohol dependence. Specifically, as the mechanism of increased proliferation would be either a shortened (accelerated) cell cycle or activation of a larger number of NPCs out of quiescence, we screened for cell cycle effects and examined which subtype of progenitor cells were proliferating at 7 days of abstinence.

## Materials and Methods

### Animal Model

**Figure 1:**
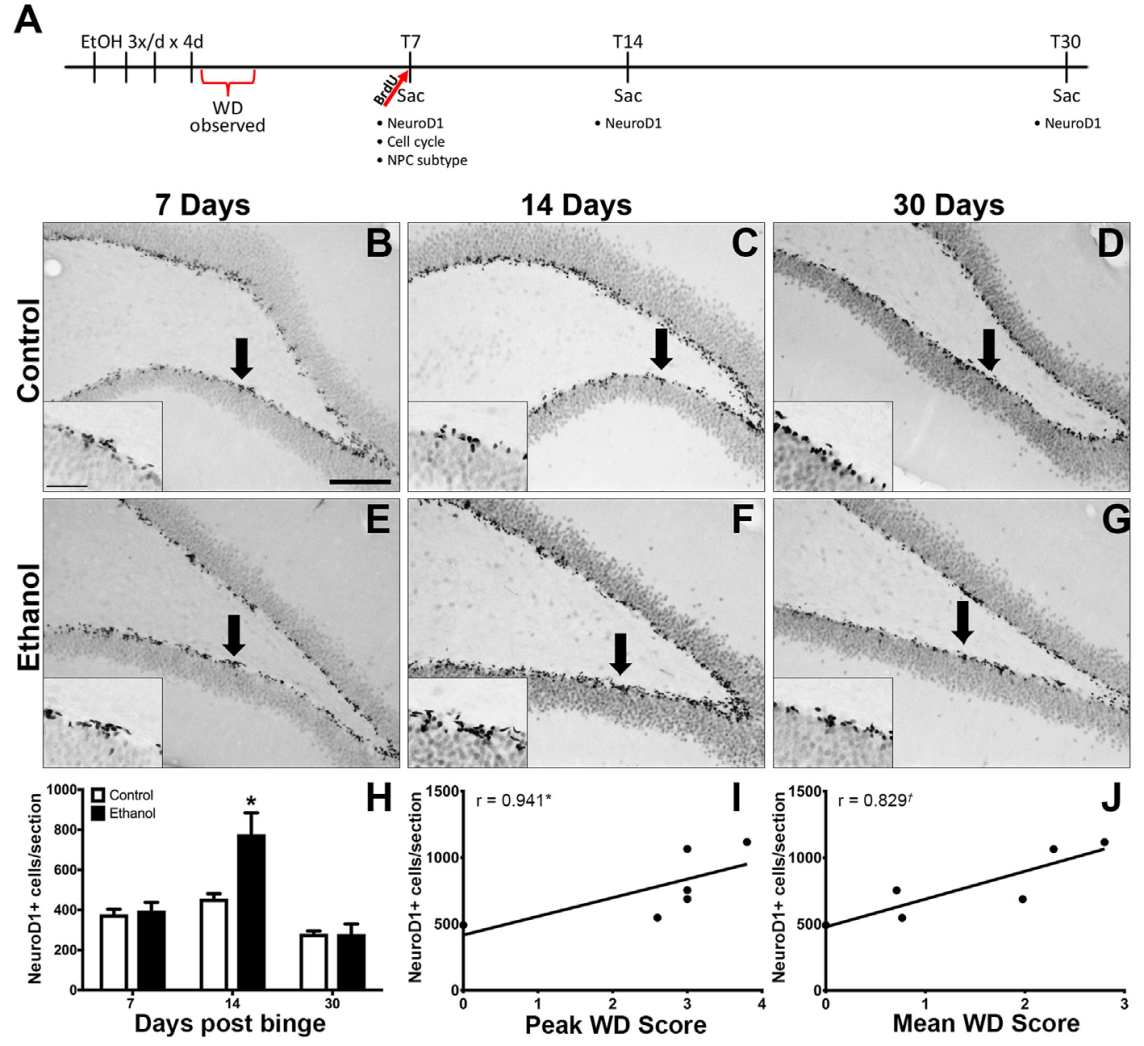
Reactive Neurogenesis confirmed with NeuroD1. **(A)** Experimental timeline is shown. Increased proliferation along the subgranular zone (SGZ) at T7 (67) is followed by enhanced NeuroD1 expression. **(B–G)** Representative images show NeuroD1 immunoreactivity present along the inner side of the granule cell layer in control **(B–D)** and ethanol **(E–G)** rats after 7, 14, and 30 days post the final dose of alcohol. Arrows point to areas represented in insets. Scale bars = 100 µm. **(H)** Profile counts revealed that the number of NeuroD1^+^ cells located in the SGZ increased significantly 14 days after binge ethanol exposure. **(I,J)** Spearman’s correlation shows a positive relationship between 14-day NeuroD1^+^ cell counts and peak withdrawal score **(I)** and mean withdrawal score **(J)**. *p < 0.05. ^†^*p* = 0.058.

Sixty-two adolescent male Sprague-Dawley rats (Charles River Laboratories; *n* = 32 controls; *n* = 30 ethanols) were used in this study. A timeline of experimental events is shown in Figure 1A. Upon arrival, postnatal day (PND) 30, rats were individually housed and allowed 5 days to acclimate to an AALAC accredited vivarium at the University of Kentucky with a 12 h light (0700)/dark (1900) cycle. All procedures were approved by the University of Kentucky’s Institutional Animal Care and Use Committee and conformed to the Guide for the Care and Use of Laboratory Animals (78).

The 4-day binge model, based on that originated by Majchrowicz (79) was chosen as it uses the common route of consumption in humans, it mimics a binge-bender typical of the truly problematic portion of the AUD population and has high BECs typical of binge-pattern drinking within the range of that reported in adolescents (80). Starting on PND 35, mid adolescence (81), rats were orally gavaged every 8 h for 4 days with either 25% w/v ethanol or isocaloric dextrose in Vanilla Ensure Plus™ that followed a procedure modified from Majchrowicz (79) as described previously (82). Rats received an initial 5 g/kg dose of ethanol with subsequent doses titrated based on the following behavioral intoxication scale: 0-normal rat (5 g/kg), 1-hypoactive (4 g/kg), 2-ataxic (3 g/kg), 3-delayed righting reflex (2 g/kg), 4-loss of righting reflex (1 g/kg), and 5-loss of eye blink reflex (0 g/kg). Control rats were given the average volume of isocaloric diet administered to the ethanol group. Three ethanol rats and one control died as a result of gavage error and/or treatment (not included in the *n* = 62), leading to unequal group numbers. Tail blood was collected 90 min after the seventh dose of ethanol diet, which is midway of the 12 total doses as well as when the peak BECs occur (82). BECs were analyzed using an AM1 Alcohol Analyser (Analox Instruments LTD., London, UK) with a 300 mg/dl standard.

Ten hours after the last dose of ethanol, animals underwent monitored withdrawal. Rats were observed for behavioral signs of alcohol withdrawal for 30 min of every hour, for 17 h exactly as reported previously (82). Animals were scored according to an established rubric of behavioral signs of withdrawal modified from Majchrowicz (79) as described previously (82, 83). Each hour the highest observed score was recorded and was then averaged across all 17 h of withdrawal (“mean WD”). For each animal, the maximum withdrawal score each rat achieved was reported as “peak WD” score.

### Tissue Collection

Based on our previous studies on reactive cell proliferation (64, 67, see text footnote 1) and important timelines in adult neurogenesis, the thymidine analog, 5-Bromo-2′-deoxyuridine (BrdU, 300 mg/kg;Roche) was injected at 2 h prior to sacrifice at 7 (T7), 14 (T14), or 30 (T30) days after their last dose of ethanol to detect changes in cell proliferation. The dose of BrdU and 2 h exposure was chosen to maximally label cells in S-phase in adolescent rats based on estimates of its half-life at around 30 min (46, 84). Rats were overdosed with sodium pentobarbital (Nembutal^®^; MWI Veterinary Supply, Nampa, ID, USA, or Fatal-Plus^®^; Vortech Pharmaceuticals, Dearborn, MI, USA) followed by transcardial perfusion using 0.1 M phosphate-buffered saline (PBS; pH 7.4) and 4% paraformaldehyde. Brains were extracted, postfixed in paraformaldehyde for 24 h and then stored in PBS at 4°C. Brains were sliced coronally into 40 µm sections with a vibrating microtome (Leica Microsystems, Wetzlar, Germany) using unbiased tissue collection methodologies. Twelve equally spaced series of sections (every 12th section) were collected beginning at a random starting point around Bregma 1.6 through approximately Bregma 6.3. Sections were stored in a cryoprotectant at −20°C until immunohistochemistry (IHC) was performed. Brains were coded so that the experimenter was blind to treatment conditions at all times.

### Immunohistochemistry

#### 3 ,3′-Diaminobenzidine Tetrahydrochloride (DAB) Labeled IHC

For antibodies to the neurogenesis-related and cell cycle-related markers, adjacent sections of every 12th (Ki67, pHisH_3_, and NeuroD1) or 6th (BrdU) tissue section were processed for free-floating IHC. To examine the number of cells in each phase of the cell cycle and calculate the percentage of cells in G_1_, S, and G_2_/M phases of the cell cycle, the following combination of cell cycle markers was measured: (1) Ki67, expressed during all stages of the cell cycle, was used to determine the number of actively dividing cells in the SGZ (85, 86); (2) BrdU, which is incorporated into the DNA during DNA synthesis [S-phase; (87)], was used to quantify cells in S-phase; (3) pHis-H_3_ was used to quantify the number of cells in G_2_ and M [G_2_/M-phase; (88)]; (4) the population of dividing cells in G_1_ phase was estimated by subtracting the total number of pHis-H_3_^+^ and BrdU^+^ cells from the number of Ki67^+^ cells. Minichromosome maintenance 2, typically used to identify G_1_ phase cells, was not specific for G_1_ phase in our hands (not shown). Thus, sections were rinsed in Tris-buffered saline (TBS) to remove traces of the cryoprotectant and incubated in 0.6% H_2_O_2_ for 30 min to quench endogenous peroxidase activity. An antigen retrieval step in Citra^®^ buffer (BioGenex, Freemont, CA) at 65°C (1 h for Ki67 or 20 min for NeuroD1 and pHisH_3_) was followed by washes in TBS then sections were blocked in 3–10% normal serum for 30 min. For BrdU, DNA-denaturing steps were included as previously described (55, 64, 77). Sections were then incubated in primary antibody for 1–2 nights at 4°C (refer to Table 1). Tissue was then washed in blocking buffer, incubated for 1 h in secondary antibody (1:200; Table 1), incubated in avidin-biotin-peroxidase complex (Vector Laboratories, Burlingame, CA, USA) for 1 h, and colorized with nickel enhanced DAB (Polysciences, Waltham, MA, USA) as previously described (55, 64, 77). Sections were mounted onto glass slides and BrdU and Ki67 were counterstained with cresyl violet and neutral red, respectively. Slides were coverslipped using Cytoseal^®^ mounting media (Richard Allen Scientific, Kalamazoo, MI, USA).

**Table 1:** Antibodies.

Primary antibody	Antibody concentration; source, product number	Incubation period (h)	Secondary antibody
**DAB (individual)**			
Mouse α-Ki67	1:200; Vector, VP-K452	48	Horse α-mouse, Vector
Rabbit α-pHisH3	1:1,000; Millipore, 06-570	16	Goat α-rabbit, Vector
Mouse α-BrdU	1:5,000; Millipore, MAB3424	16	Horse α-mouse, Vector
Goat α-NeuroD1	1:1,000; Santa Cruz, sc-1054	48	Rabbit α-goat, Vector

**Fluorescent**			
Double			
Sox2	1:200; Millipore, AB5603	48	AlexaFluor goat α-rabbit 488, Invitrogen
BrdU	1:400; Accurate; OBT0030	48	AlexaFluor goat α-rabbit rat 546, Invitrogen
Quad			
Mouse α-Ki67	1:100; Vector, VP-K452	96	Donkey α-mouse 405, Jackson ImmunoResearch
Chicken α-GFAP	1:1,000; Abcam, ab467m	96	Donkey α-chicken 488, Jackson ImmunoResearch
Goat α-NeuroD1	1:500; Santa Cruz, sc-1054	96	Donkey α-goat 633, Invitrogen
Rabbit α-Sox2	1:200; Millipore, AB5603	96	Donkey α-rabbit 546, Invitrogen

#### Fluorescent IHC

In order to examine the number of proliferating NPCs or differentiate type 1, 2a, 2b versus 3 progenitor cells, a series of every 12th section of T7 tissue was processed for double (Sox2^+^/BrdU^+^) or quadruple (Ki67, GFAP, Sox2, and NeuroD1) fluorescent IHC as described (see text footnote 1). Briefly, tissue was washed in TBS, followed by antigen retrieval steps [BrdU: DNA denaturing as in Ref. (64), Quad label: sodium citrate buffer at 65°C for 1 h]. Sections were washed, blocked in 3% or 10% normal serum, and incubated in primary antibodies (Table 1) for 48 h (double) or 96 h (quad). Sections were then rinsed in blocking buffer and incubated in fluorescent secondary antibody for 1 h (double) or overnight (quad) in the dark (Table 1). Following additional washes in TBS, sections were mounted onto glass slides, dried, and coverslipped with ProLong^®^ Gold anti-fade reagent (Life Technologies, Eugene, OR, USA).

### Quantification of IHC

#### DAB-Based IHC

The number of immunoreactive cell profiles (BrdU, Ki67, NeuroD1, and pHisH_3_) within hippocampal SGZ were quantified using a 100x objective and an Olympus BX-41 microscope (Olympus, Center Valley, PA). A profile counting approach was chosen over stereology for several reasons besides expediency: (a) the question of interest is relative difference versus controls which we have previously shown to be identical for profile counts versus stereology for proliferation markers (89), (b) stereology is not appropriate for proliferation markers as they are heterogeneously scattered along the SGZ and relatively few in number (90), and (c) the volume of the hippocampus is not different between ethanol and controls (91). The SGZ was defined as a ~50 μm thick ribbon of tissue between the granule cell layer and hilus of the dentate gyrus. As tissue is collected in an unbiased procedure, immunopositive profiles were counted across 6–8 sections (every 12th) or 8–10 sections (every 6th) per brain and presented as mean number of immunopositive profiles ± SEM.

#### Double Fluorescent-Labeled IHC

Colabeled BrdU^+^ and Sox2^+^ cells were quantified along the SGZ using a 100x objective lens with an Olympus BX51 microscope (Olympus, Center Valley, PA, USA) with epifluorescence and bandpass filter cubes to visualize red (546 nm) and green (488 nm). Similar to above, as tissue was collected in an unbiased procedure, colabeled cells were counted across six to eight sections per brain as follows: Analysis started with BrdU^+^ cells, which were then evaluated for the presence or absence of Sox2 expression and reported as the mean number of colabeled cells per section ± SEM.

#### Quadruple Fluorescent-Labeled IHC

Sox2 labels multiple types of progenitor cells and the subtypes respond differently to neurogenic stimuli (92, 93). To determine the subtypes of NPCs responding during the proliferation burst at T7, a quadruple fluorescent IHC scheme was devised to differentiate proliferating type 1, 2a, 2b, and 3 cells simultaneously in tissue (see text footnote 1). To identify type 1, 2a, 2b, and 3 progenitor cells, a Leica TCS SP5 confocal microscope (Wetzlar, Germany) was used to collect z-stack images of 40 cells across five to six sections per brain under a 63.4x lens at 0.8 µm thickness, similar to previous (60, see text footnote 1). Proliferating cells (Ki67^+^) were defined as type 1 (GFAP^+^/Sox2^+^/NeuroD1^−^), type 2 (type 2a = GFAP^−^/Sox2^+^/NeuroD1^−^; type 2b = GFAP^−^/Sox2^+^/NeuroD1^+^), and type 3 (GFAP^−^/Sox2^−^/NeuroD1^+^) according to published definitions identical to our previous work in adults (61, 92, see text footnote 1). Cells were evaluated for colabeling in z-stack images rendered into a 3D model by ImagePro Plus 3D software (6.3, Media Cybernetics, Silver Springs, MD, USA). Due to software limitations, only three channels could be compared simultaneously. Therefore, two separate 3D renderings were made for each z-stack. The first included NeuroD1, Sox2, and Ki67 and were used to quantify type 2a, 2b, and 3 NPCs (Figure 4A). The second included GFAP, Sox2, and Ki67 and were combined with data collected from the first rendering to differentiate type 1 from type 2a progenitors. Each channel’s surface values were adjusted to minimize background signal while maintaining visibility of the fluorescent immunoreactivity. To ensure accuracy, the 3D renderings were compared side by side with the raw z-stack images during quantification. The percentage of cells of each subtype ± SEM is presented along with an estimate of the number of proliferating cells generated by multiplying the percentages obtained with actual counts of DAB-labeled Ki67^+^ cells.

### Statistics

All data were initially assembled in Microsoft Excel with statistical tests performed using either Prism (GraphPad, LaJolla, CA, USA) or SPSS (IBM, Version 22, Armonk, NY, USA) software. Data are graphed as mean ± SEM. BECs and mean ethanol dose per day were analyzed by one-way analysis of variance (ANOVA) followed by *post hoc* Tukey’s tests. Intoxication and withdrawal behavior scores were analyzed by the non-parametric Kruskal-Wallis. Histological data were analyzed by appropriate ANOVA followed by Bonferroni *post hoc* tests. Correlation between histology and withdrawal behavior was assessed by the non-parametric, Spearman correlation. *p*-values were accepted as significantly different at *p* < 0.05.

## Results

### Binge Data

Ethanol intoxication parameters including mean intoxication scores, daily ethanol dose, BECs, and mean and peak withdrawal scores for each cohort are presented in Table 2. While all binges were conducted identically, groups occasionally differ in some parameters. Table 2 illustrates that the mean BECs [*F*_(3,27)_ = 4.17; *p* < 0.05] and mean WD scores [*F*_(3,29)_ = 3.09; *p* < 0.05] were significantly lower in the animals at the T30 timepoint (30 days postbinge alcohol exposure) than those in the T14 group (14 days postbinge). Despite the T30 group having a lower BEC, the average daily dose of ethanol was significantly higher compared to T14 [*F*_(3,29)_ = 4.73; *p* < 0.001]. T14 animals also received a lower mean dose per day than T7 group 2 (*p* < 0.05), reflecting the increased intoxication scores of the T14 group [*F*_(3,29)_ = 6.95; *p* < 0.005]. Despite higher BEC’s and mean WD scores in the T14 group, there was no difference between T14 and T30’s intoxication score. The variable dosing in this model is to maintain high blood alcohol levels (>200 mg/dl) across the 4 days of alcohol exposure, which these measures confirmed did occur. Importantly, all values were within the range previously reported for this model (82).

**Table 2:** Binge intoxication parameters.

Time point (days post EtOH)	*n*	Intoxication score	EtOH dose (g/kg/day)	BEC (mg/dL)	Mean withdrawal	Peak withdrawal
T7 (1-Quad label)	8	1.0 ± 0.1	11.9 ± 0.4	372.5 ± 18.7	1.0 ± 0.3	3.1 ± 0.3
T7 (2—all other)	7	0.7 ± 0.1	13.0 ± 0.3	363.3 ± 21.7	1.2 ± 0.2	3.5 ± 0.1
T14	6	1.4 ± 0.1^a^	10.9 ± 0.3^a^	388.6 ± 22.0	1.4 ± 0.4	2.6 ± 0.5
T30	9	1.0 ± 0.1	12.3 ± 0.2^b^	309.7 ± 11.7^b^	0.3 ± 0.1^b^	2.1 ± 0.3

*All controls n = 8*.

*^a^p < 0.05 vs. T7 group 2*.

*^b^p < 0.05 vs. T14*.

*EtOH, ethanol; BEC, blood ethanol concentration*.

### Reactive Neurogenesis Confirmed with NeuroD1

Our prior report on reactive adult neurogenesis after 4-day binge ethanol exposure in adolescent rats utilized Doublecortin expression to identify immature neurons (67). As Doublecortin may not be specific for newborn neurons (94), NeuroD1 IHC was used to identify late stage progenitor cells committed to a neuronal fate (95). NeuroD1 immunoreactivity was observed in a distinct line along the dentate gyrus SGZ in all groups as expected (Figures 1B–G). In the T14 group, those ethanol-exposed rats with the most severe withdrawal scores also had ectopic expression of NeuroD1^+^ cells in the hilus and molecular layer of the dentate gyrus (data not shown) as expected based on our prior report of ectopic Doublecortin and Prox-1 expression in high withdrawal severity adolescent rats only (67). The number of NeuroD1^+^ cells was counted along the SGZ only at T7, T14, and T30 days following 4-day binge ethanol exposure and reported as mean cells per section (Figure 1H). A two-way ANOVA (diet x time point) revealed significant main effects of diet [*F*_(1,40)_ = 11.35, *p* < 0.005], time [*F*_(2,40)_ = 34.29, *p* < 0.001], and a significant diet × time interaction [*F*_(2,40)_ = 9.24, *p* < 0.001]. A *post hoc* Bonferroni test for multiple comparisons showed that the number of NeuroD1^+^ cells was significantly increased in the ethanol-treated group at T14 versus its respective control [*F*_(1,12)_ = 11.34, *p* < 0.01]. There was no difference in the number of NeuroD1^+^ cells between ethanol and control rats at 7 (T7) or 30 (T30) days postbinge. Next, we examined the relationship between NeuroD1 expression at T14 and ethanol withdrawal severity, similar to our previous report (67). The results showed a positive relationship between the number of NeuroD1^+^ cells at T14 and peak withdrawal score (*r* = 0.941; *p* = 0.017, Figure 1I), and mean withdrawal score (*r* = 0.829; *p* = 0.058, Figure 1J).

### Cell Cycle Distribution in Adolescent Rats during Early Abstinence

Alcohol-induced reactive neurogenesis originated, in part, from a striking burst in cell proliferation at T7 of abstinence in the adolescent rat (67). Such increases in proliferation are due to either an increase in the number of proliferating progenitor cells and/or an acceleration (shortening) of the cell cycle. As we previously identified that alcohol accelerates the cell cycle during intoxication with 4 days of binge alcohol exposure in adolescent male rats (77), we screened for cell cycle effects remaining 7 days later, though in abstinence. The screen is sensitive to changes in the cell cycle based on the expression of various cell cycle specific markers, but uses a much smaller number of animals than is required for the saturate and survive methods used to study cell cycle kinetics (96).

**Figure 2:**
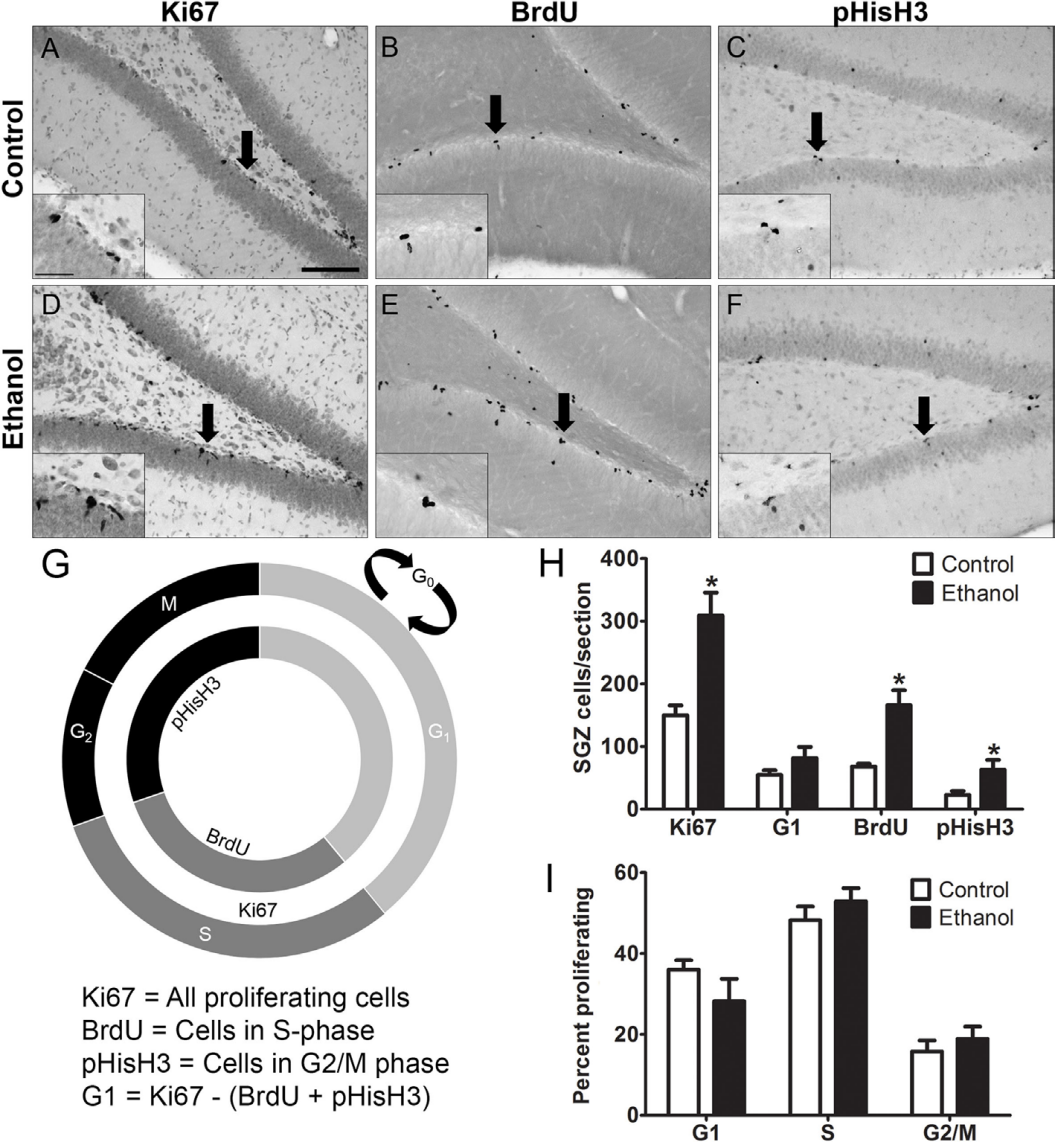
Binge ethanol exposure during adolescence and cell cycle distribution of subgranular zone neural progenitor cells (NPCs) at day 7 of abstinence. **(A–F)** Representative images from sections stained for Ki67, BrdU, and pHis-H_3_. Arrows denote area represented in the inset. **(G)** Cell cycle diagram showing the stages of the cell cycle labeled by Ki67, BrdU, and pHis-H_3_. BrdU labels cells in S-phase, pHis-H_3_ labels cells in G_2_ and M phase, and Ki67 labels actively dividing cells of all stages. G_1_ population is calculated by subtracting total BrdU^+^ and pHis-H_3+_ cells from Ki67^+^ cell numbers. **(H)** Quantification data of dividing cells in Ki67^+^ (total), BrdU^+^ (S phase), and pHisH_3_^+^ (G_2_/M) cells. Calculated number of cells in G_1_ was obtained by subtracting the number of BrdU^+^ and pHisH_3_^+^ cells from the number of Ki67 cells. **(I)** Calculated distribution of dividing NPCs within each phase of the cell cycle based on total number of Ki67 cells.

Representative photomicrographs show that clusters of Ki67^+^, BrdU^+^, and pHisH_3_^+^ cells were visible along the SGZ of the dentate gyrus (Figures 2A–F). Similar to previous work (67), ethanol animals showed a 2-fold increase in the number of Ki67^+^ cells compared to controls [*F*_(1,14)_ = 15.934, *p* = 0.001], a 2.5-fold increase in the number of BrdU^+^ cells compared to controls [*F*_(1,12)_ = 15.382, *p* < 0.01], and a 2.4-fold increase in the number of pHis-H_3_^+^ cells compared to controls [*F*_(1,14)_ = 4.655, *p* < 0.05]. The calculated number of cells in G_1_ phase [i.e. G_1_ = Ki67^+^ – (BrdU^+^ + pHisH_3_^+^)] was only slightly but not significantly higher in the ethanol rats versus controls [*F*_(1,14)_ = 1.931, *p* = 0.186; Figure 2H]. Next, to determine the effect of alcohol on the distribution of cells across each phase of the cycle (detailed in Figure 2G), the proportion of cells within G_1_, S, and G_2_/M of all actively cycling hippocampal NPCs was calculated (Figure 2I). The results show that 7 days after binge alcohol exposure there were no changes in the proportion of hippocampal NPCs in each cell cycle phase in adolescent rats (Figure 2I), which suggests that the cell cycle was not altered by prior ethanol exposure at this time point (T7), similar to that observed in adult rats (see text footnote 1).

### Characterization of Proliferating Progenitors

**Figure 3:**
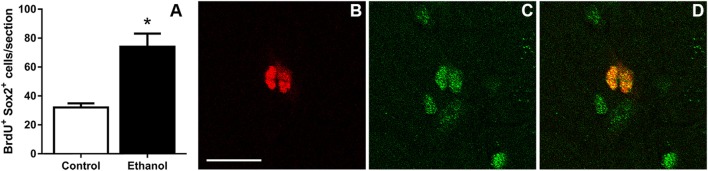
Binge ethanol exposure during adolescence increases the number of subgranular zone neural progenitor cells at day 7 of abstinence. **(A)** Quantification data of BrdU^+^ and Sox2^+^ co-positive cells in control and alcohol rats. **(B–D)** Representative fluorescent images for BrdU [red **(B)**] and Sox2 [green **(C)**] and colabel **(D)**. Scale bars = 100 µm. *p < 0.05.

The similar fold increase in the number of Ki67^+^, BrdU^+^, and pHis-H_3_^+^ cells supported that binge ethanol exposure in adolescent rats activates hippocampal NPCs and leads to NPC proliferation. This reactive proliferation may be due to an expansion of the proliferating progenitor pool. Therefore, to test this hypothesis, the number of proliferating progenitor cells was examined by exhaustively counting the number of BrdU^+^/Sox2^+^ colabeled cells in the SGZ. Sox2^+^ and BrdU^+^ cells lined the SGZ as expected and similar to past work [data not shown; see text footnote 1]. The number of BrdU^+^ cells copositive for Sox2 was counted in each group and ethanol-exposed rats showed a significant twofold increase in the number of BrdU^+^/Sox2^+^ cells at T7 compared to controls [*F*_(1,12)_ = 16.6, *p* < 0.005; Figure 3]. The magnitude of this increase was similar to BrdU alone and confirmed that, at the T7 time point in male adolescent rats, the proliferating cells were NPCs.

**Figure 4:**
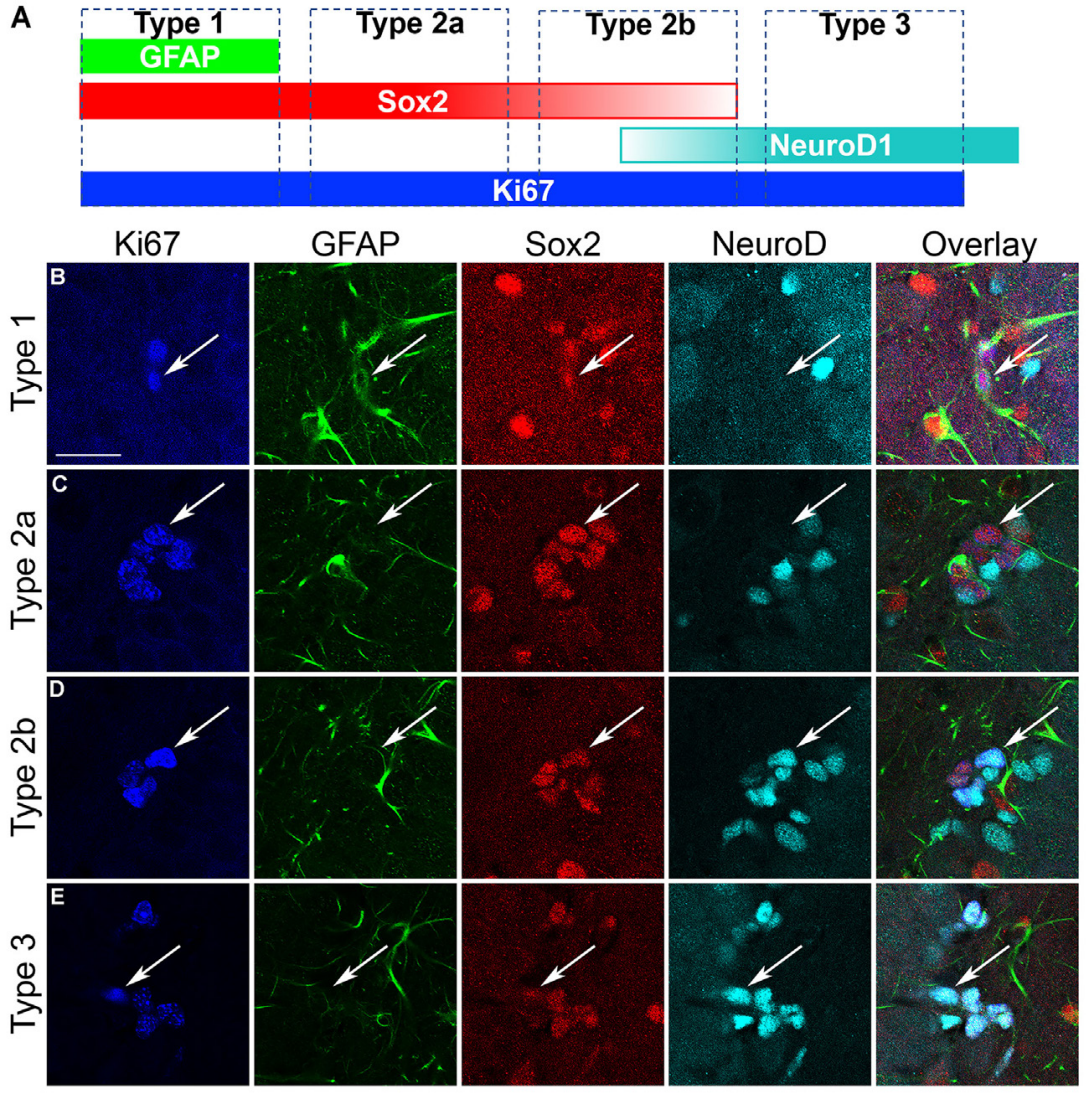
Characterization of hippocampal neural progenitor cells. **(A)** Quadruple fluorescent immunohistochemistry was applied to identify each of the various progenitor cell subtypes as defined in the schematic as follows: GFAP^+^/Sox2^+^/NeuroD1^−^ cells are considered as type 1; GFAP^−^/Sox2^+^/NeuroD1^−^ cells are considered as type 2a; GFAP^−^/Sox2^+^/NeuroD1^+^ cells are considered as type 2b; GFAP^−^/Sox2^−^/NeuroD1^+^ cells are considered as type 3 cells. **(B–E)** Representative confocal images of each of the subtypes show Ki67^+^ cells colabeled with GFAP, Sox2, and/or NeuroD1 according to that defined in **(A)**. Scale bars = 20 µm.

**Figure 5:**
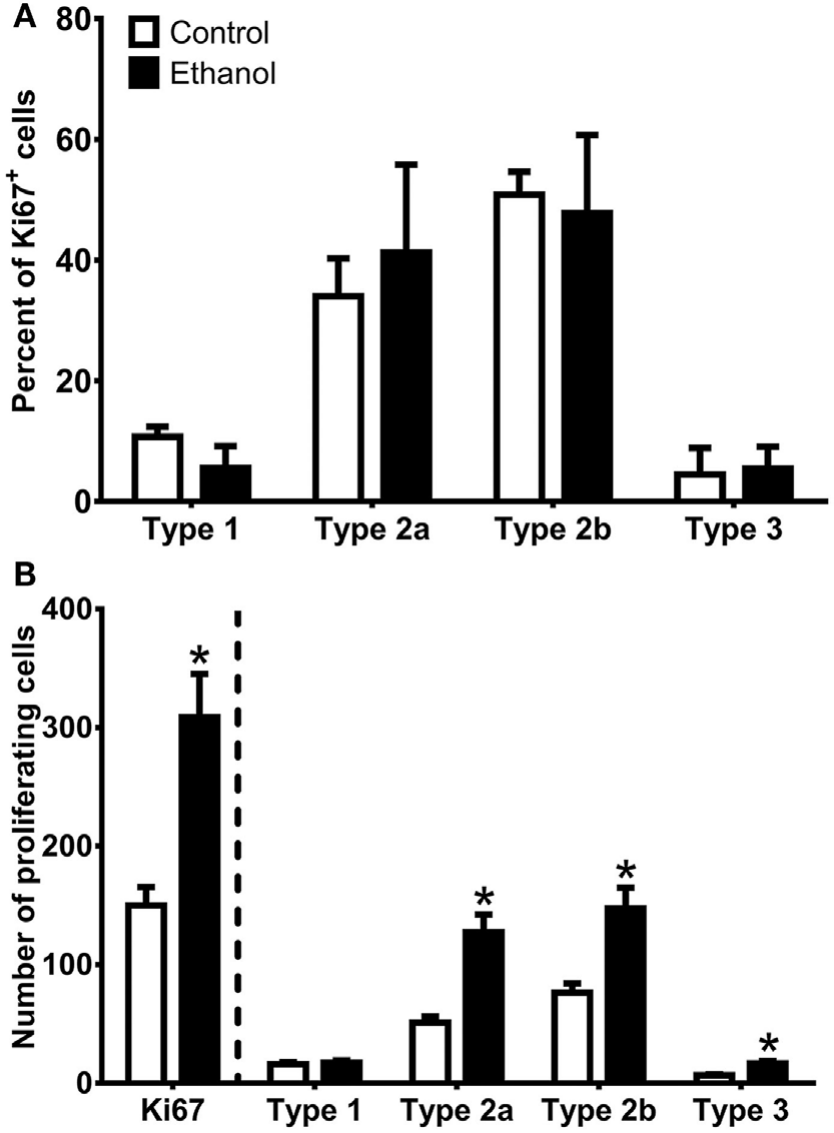
Quantification of progenitor cell subtypes at day 7 of abstinence. **(A)** The graph shows the proportion of sampled Ki67^+^ cells that were type 1 (GFAP^+^/Sox2^+^/NeuroD1^−^), type 2a (GFAP^−^/Sox2^+^/NeuroD1^−^), type 2b (GFAP^−^/Sox2^+^/NeuroD1^+^), and type 3 (GFAP^−^/Sox2^−^/NeuroD1^+^). **(B)** The graph shows calculated NPC subtypes based on the number of Ki67^+^ cells present in the SGZ multiplied by the NPC subtype proportions. * indicates *p* < 0.05.

As Sox2 labels multiple types of progenitor cells, a quadruple fluorescent IHC scheme was devised to differentiate proliferating type 1, 2a, 2b, and 3 cells simultaneously in tissue (Figure 4A; see text footnote 1). Thus, 40 Ki67^+^ cells (cells in active cycle) for each rat hippocampus were examined for colabeling with GFAP, Sox2, and NeuroD1 in 3D renderings of Z-stacks obtained from a confocal microscope. Representative confocal images for each subtype is presented in Figure 4. Quadruple-label immunofluorescence for Ki67/GFAP/Sox2/NeuroD1 IHC demonstrated that the majority of cells were type 2 (type 2a = GFAP^−^/Sox2^+^/NeuroD1^−^; type 2b = GFAP^−^/Sox2^+^/NeuroD1^+^) with low percentages of type 1 cells (GFAP^+^/Sox2^+^/NeuroD1^−^) and type 3 cells (GFAP^−^/Sox2^−^/NeuroD1^+^) as expected (61, 92, see text footnote 1). No differences between control and ethanol groups were observed in the proportion of all four subtypes (type 1, 2a, 2b, 3; Figure 5A) as analyzed by one–way ANOVA. Next, the number of cells in each of the four subtypes was calculated: *n*, the number of Ki67^+^ cells in the SGZ (Figure 2H) was multiplied by the cell subtype proportions (in 5 A). The twofold increase in the number of Ki67^+^ cells resulted in similar significant increases in the numbers of type 2a, 2b, and 3 cells in ethanol-treated rats compared with controls according to one-way ANOVAs [type 2a: *F*_(1,15)_ = 22.79, *p* < 0.001; type 2b: *F*_(1,15)_ = 13.79, *p* < 0.005; and type 3: *F*_(1,15)_ = 23.01, *p* < 0.001]. There was no significant difference in the number of type 1 cells between control and ethanol-exposed rats (Figure 5B). Thus, type 2a cells were activated into the cell cycle as expected (92) but there were also significantly more proliferating type 2b and 3 cells that underlie reactive neurogenesis in abstinence.

## Discussion

In this study, we demonstrate that adolescent rats exhibit reactive hippocampal neurogenesis after 4-day binge ethanol exposure, confirmed by the enhanced expression of the immature neuronal marker, NeuroD1, 14 days after ethanol exposure (Figure 1). As previous work (67) demonstrated that reactive neurogenesis originated with an increase in hippocampal cell proliferation at 7 days following 4-day binge ethanol exposure, we examined two potential mechanisms of this increase: either *via* a shortened (accelerated) cell cycle or activating a larger number of NPCs out of quiescence and into the cell cycle. First, we investigated the effect of prior ethanol exposure on the number and distribution of hippocampal NPCs across the G_1_, S, and G_2_/M phases of the cell cycle. Prior binge alcohol exposure significantly increased NPC cell numbers in S and G_2_/M phases (G_1_ was increased, but not statistically) without changing the proportion of cells in each phase (Figure 2I). Therefore, the effects of alcohol on the number of cells in S and G_2_/M phases was more likely due to an increase in the number of actively cycling cells. These data ruled out an accelerated (shortened) cell cycle underlying alcohol-induced reactive neurogenesis in adolescent rats. Next, we showed that the reactive increase of cell proliferation seven days after alcohol exposure in adolescent rats was in actively proliferating NPCs, evidenced by a twofold increase in the number of BrdU^+^/Sox2^+^ colabeled cells (Figure 3). As Sox2 is expressed in multiples subtypes of progenitors (93) we probed further to examine whether prior alcohol affected any subtype of progenitor differentially. A quadruple fluorescent labeling scheme to differentiate proliferating type 1, 2a, 2b versus 3 cells revealed that prior alcohol exposure did not alter the percentage of cells classified as any of the four subtypes, but did increase the estimated numbers of proliferating type 2a, 2b, and 3 cells (Figure 5). These data support that alcohol-induced reactive neurogenesis is due to prior alcohol dependence, or its sequelae, activating NPCs out of quiescence and into active cycling at day 7 (T7) of abstinence.

The first experiment examined the number of NeuroD1^+^ cells as our prior reports on reactive neurogenesis used Doublecortin, the former gold standard marker for neuroblasts, though recently observed in oligodendrocyte progenitors (94, 97, 98). NeuroD1, a basic helix-loop-helix transcription factor necessary normal neuronal development (95, 99–101), has an expression profile very similar to Doublecortin; it is expressed in mid- to late-stage NPCs committed to a neuronal cell fate (102). A further benefit of NeuroD1, as it is a transcription factor as opposed to the microtubule-associated protein, Doublecortin, NeuroD1 has a nuclear pattern of immunoreactivity and is therefore easier to quantify with profile cell counts or colabeling analysis of cell phenotype. At T14, the increased number of NeuroD1^+^ cells along the SGZ in ethanol rats compared to control rats followed the increase in proliferation at T7, a pattern identical to that reported previously for Doublecortin immunoreactivity in both adult and adolescent rats exposed to the 4-day binge ethanol model (64, 67, see text footnote 1). Ectopic NeuroD1^+^ cells were also observed as expected from our previous report of ectopic Doublecortin in the molecular and hilus layers (67). Ectopic NeuroD1 was not quantified for the current report as this work focuses on the progenitor cells of the SGZ. As adult born granule cells do not become fully integrated into existing hippocampal circuitry until 4–8 weeks following birth (103, 104) and the increased NeuroD1^+^ cells were observed at only 2 weeks post ethanol, additional work should determine if these newly generated “reactive” neurons integrate properly into the existing hippocampal circuitry.

Next (Figure 3), we determined that cells proliferating in the SGZ, indicated by immunoreactivity for the S-phase marker, BrdU, were proliferating NPCs. We observed an increase in the number of cells colabeled for Sox2 and BrdU in the SGZ in the ethanol group as compared to controls, which supports that prior alcohol dependence results in an increase in the number of proliferating NPCs. As Sox2 labels multiple subtypes of proliferating NPCs (93) and each of these subtypes respond distinctly to neurogenic stimuli [e.g., (92)], we hypothesized that the type 2a progenitor would respond robustly. Our results show that increases in proliferation are largely seen in type 2 cells, in agreement with work that this cell type rapidly proliferates to neurogenic stimuli (92). Both the type 1 and type 3 cells generally accounted for less than 5% of the proliferating pool of cells, similar to our observations in adult rats (see text footnote 1). The lack of alcohol effect on the number of proliferating type 1 cells at T7 could be rooted in the low number of type 1 progenitors that actively proliferate coupled with our random sampling of 40 Ki67^+^ cells. As such, a limitation in our approach is that only cells immuno-labeled with Ki67 are assessed and Ki67 may be undetectable during portions of early G_1_ phase (86). Additionally, prior alcohol could theoretically affect the expression of Ki67. However, in adults, type 1 cells are recruited out of quiescence to a greater extent in 4-day binge alcohol rats as opposed to controls at this same time point (see text footnote 1), an observation that mirrors that seen in other brain insults (76, 105–107). Furthermore, only one time point in abstinence after alcohol dependence was assessed. In adults, NPC proliferation begins as early as T5 with only type 2 progenitors activated as predicted, though progressing to all four types by T7 (see text footnote 1). Therefore, different populations of NPCs could be activated into the cell cycle in a time line distinct from adults and should be assessed in future studies. Activation of different pools of progenitors has implications for mature neuronal phenotypes that arise from these progenitors (108).

A previous study from our laboratory in the same 4-day binge model demonstrated that ethanol intoxication specifically reduces the length of the S-phase in hippocampal NPCs without altering the G_1_ or G_2_/M phases (77). Utilizing the same screening approach as employed above, it was clear that the cell cycle was affected (BrdU^+^ cells reduced, while Ki67^+^ cells were the same between adolescent alcohol and controls). Thus, the positive screen justified full study of cell cycle kinetics using the cumulative BrdU injection method (87). At T0, which is during intoxication, immediately after the last dose of alcohol in the 4-day binge, alcohol reduced NPC cell cycle duration by 36% and shortened S-phase by 62%, suggesting that binge alcohol exposure accelerates NPC cell cycle progression in adolescent rats (77). This acceleration resulted in an expansion of the NPC pool as indicated by a significant increase in the number of Sox2^+^ NPCs in the hippocampal SGZ immediately following binge alcohol exposure. Therefore, 4-day binge ethanol intoxication in adolescent rats, specifically, shortens cell cycle length [at T0; (77)] which should increase the NPC pool, which is exactly what we then detected at T7 of abstinence (Figures 3 and 5). Interestingly, the cell cycle appears to return to control levels as cells were in similar proportions across the phases of the cell cycle for both prior ethanol exposed and control rats (Figure 2).

Neural progenitor cells along the SGZ of the hippocampus continuously generate new granule neurons throughout life, a phenomenon critical to hippocampal structure and function, namely, hippocampal-dependent learning and memory (45, 48, 109). Increases in adult neurogenesis are associated with improved hippocampal functions such as learning, memory, and mood (45, 49, 50, 110–112). Reactive neurogenesis and/or activation of NPCs after insult also contributes to recovery in other models of CNS insult (113–116). However, reactive neurogenesis in seizure appears to contribute to epileptogenesis (74, 75). Therefore, as alcohol dependence in adolescence results in withdrawal seizures in some animals (82), it is not known whether reactive neurogenesis after alcohol dependence is a beneficial repair mechanism or a pathological phenomenon (117). Data support both sides: reactive neurogenesis after alcohol dependence in adult rats correlates to recovery of dentate gyrus granule cell number (see text footnote 1) but reactive neurogenesis in adolescents can be ectopic if withdrawal is severe, similar to the ectopic new neurons observed in seizure models (67, 74). As speculated in Ref. (67), ectopic neurogenesis may be yet another aspect of the adolescent’s susceptibility to alcohol-induced hippocampal dysfunction as ectopic neurogenesis is thought to contribute to hippocampal pathology in epilepsy (117). Fortunately, overt signs of alcohol withdrawal are less common in adolescents than adults (118), though behavioral symptoms of severity are identical between adult and adolescent rats in the model used (82). In sum, a critical future direction is to elucidate the role of reactive neurogenesis after alcohol dependence in adolescent rats specifically.

Another important question that arises from this body of work concerns the cause of reactive neurogenesis. That reactive neurogenesis is common to many forms of CNS insult suggests that cell death may be a common trigger of the phenomenon, especially since there is significant cell death in the 4-day binge model used here (12, 119–121). However, reactive neurogenesis has been observed in milder alcohol dependence models where there is less acute cell death than in this binge model (65, 66, 68). Seizure or excitatory activity in the hippocampus also results in reactive neurogenesis and seizure is observed in some animals in this model as discussed above. Intriguingly, in adults at least, eliminating overt seizures with diazepam did not prevent reactive cell proliferation from occurring (64). Diazepam does not suppress all behaviors that result from withdrawal-induced over-excitation though (122). Therefore, residual excitatory activity could continue to drive reactive neurogenesis through the recruitment of progenitors, as in other models (123–125). Indeed, the development of alcohol dependence is due, in part, to chronic inhibition of the N-methyl-d-aspartate (NMDA) receptor (126), while alcohol dependence-induced reactive neurogenesis mirrors NMDA receptor blockade effects on NPC proliferation and neurogenesis (127, 128). Thus, alcohol dependence and specifically, alcohol withdrawal-induced hyperexcitability, likely plays a major role in reactive neurogenesis in models of AUDs (64, 67, 68).

The resulting effect of increased neurogenesis detected in abstinence clearly requires further investigation in both adult and adolescent models of AUDs. It is worthy to note that the effects described occur with one 4-day exposure. Those with AUDs do not merely binge once or become dependent once. Therefore, future studies should consider models where there are cycles of dependence and withdrawal. That reported by Somkuwar et al. (68), however, highlights that long-term dependence facilitated by cycles of ethanol vapor inhalation, induces similar effects on reactive neurogenesis. Indeed, it is the similar results in these two models, besides the very different routes to dependence, that support our conclusion that an aspect of alcohol dependence is likely the major player in reactive neurogenesis.

## Ethics Statement

All procedures were approved by the University of Kentucky’s Institutional Animal Care and Use Committee and conformed to the Guide for the Care and Use of Laboratory Animals (78).

## Author Contributions

Conceived and designed experiments (CGN, KN, and DH), conducted experiments (CGN, JM, DH, KC, and KN), analyzed and/or interpreted data (CGN, HP, KC, and KN), and drafted and/or revised document (CGN, HP, DH, KC, JM, and KN).

## Conflict of Interest Statement

The authors have no conflicts of interest to declare: this research was conducted in the absence of any commercial or financial relationships that could be construed as a potential conflict of interest.

## Footnotes

^1^Hayes DM, Geil Nickell CR, Chen KY, McClain JA, Heath MM, Nixon K. Activation of neural stem cells from quiescence drives reactive hippocampal neurogenesis after alcohol dependence. *Neuropharmacology*. (In Review).
